# Diagnostic accuracy of diffusion-weighted magnetic resonance imaging for cervical lymph node metastasis from oral cancer

**DOI:** 10.1590/0100-3984.2024.0064

**Published:** 2025-04-11

**Authors:** Suneela Shaukat, Ali Mansoor, Nawaz Rashid, Zara Shaukat, Umar Amin, Sobia Mazhar

**Affiliations:** 1 Mayo Hospital Lahore, Lahore, Punjab, Pakistan; 2 Shaukat Khanum Memorial Cancer Hospital, Lahore, Punjab, Pakistan; 3 Akhtar Saeed Medical & Dental College, Lahore, Punjab, Pakistan

**Keywords:** Lymph nodes/pathology, Mouth neoplasms, Head and neck neoplasms, Lymphatic metastasis, Diffusion magnetic resonance imaging, Sensitivity and specificity, Linfonodos/patologia, Neoplasias bucais, Neoplasias de cabeça e pescoço, Metástase linfática, Imagem de difusão por ressonância magnética, Sensibilidade e especificidade

## Abstract

**Objective:**

To determine the accuracy of diffusion-weighted imaging (DWI) for the diagnosis of cervical lymph node metastasis from oral cancer.

**Materials and Methods:**

This was a cross-sectional study conducted in the Radiology Department of the Mayo Hospital, in the city of Lahore, Pakistan. We included 150 patients diagnosed with oral cancer. Ages ranged from 18 to 60 years of age. During the study period, all of the patients included underwent magnetic resonance imaging, including a DWI sequence, in a 1.5-T scanner with a phased-array head and neck coil. Patients with contraindications to magnetic resonance (aneurysm, a pacemaker, clips, plates, a prosthetic valve, or claustrophobia) were excluded. In the DWI sequence, the area scanned included the lymph nodes from suprasternal notch to the base of the skull. Histopathology of the lymph nodes was employed as the gold standard.

**Results:**

The sensitivity, specificity, positive predictive value, negative predictive value, and accuracy of DWI for the diagnosis of oral cancer metastasis to cervical lymph nodes, with histopathology as the gold standard, was 90.57%, 91.75%, 94.68%, 90.57%, and 91.33%, respectively.

**Conclusion:**

Our findings indicate that DWI is fairly accurate for detecting metastases in the cervical lymph nodes of patients with oral cancer.

## Abstract (pt)

**Objetivo:**

Determinar a precisão da DWI para o diagnóstico de metástase de linfonodos cervicais de câncer oral.

**Materiais e Métodos:**

Trata-se de um estudo transversal realizado no Serviço de Radiologia do Mayo Hospital, na cidade de Lahore, Paquistão. Incluímos 150 pacientes com diagnóstico de câncer oral. As idades variaram de 18 a 60 anos. Durante o período do estudo, todos os pacientes incluídos foram submetidos a ressonância magnética, incluindo uma sequência DWI, em um *scanner* de 1,5-T com uma bobina de cabeça e pescoço *phased-array*. Pacientes com contraindicações à ressonância magnética (aneurisma, marcapasso, clipes, placas, válvula protética ou claustrofobia) foram excluídos. Na sequência DWI, a área escaneada incluiu os linfonodos da incisura supraesternal até a base do crânio. A histopatologia dos linfonodos foi empregada como padrão-ouro.

**Resultados:**

Os valores de sensibilidade, especificidade, valor preditivo positivo, valor preditivo negativo e precisão da DWI para o diagnóstico de metástase de câncer oral em linfonodos cervicais, com histopatologia como padrão-ouro, foram 90,57%, 91,75%, 94,68%, 90,57% e 91,33%, respectivamente.

**Conclusão:**

Nossos resultados indicam que a DWI é bastante precisa na detecção de metástases nos linfonodos cervicais de pacientes com câncer oral.

## INTRODUCTION

Oral and oropharyngeal cancers are uncommon in Western countries but have a high prevalence worldwide, being ranked as the sixth most common malignancy^(1)^. An estimated 400,000 new cases of oral or oropharyngeal malignancies are diagnosed each year^(2)^. Two thirds of those cases occur in Asian countries. There is a huge disease burden in Pakistan, where oral cancer is the most common malignancy in men^(3)^. Women in the country are also commonly affected; oral squamous cell carcinoma is the second most common malignancy among females in Pakistan^(4)^. It is associated not only with morbidity but also with high mortality. Staging of cancers is fundamental. That is the first step before any treatment (surgical or nonsurgical) can be initiated, because the choice of treatment is dependent on the stage of the disease. In the case of oral cancers, metastatic involvement of lymph nodes upstages the disease from stage 2 to stage 3. This has important implications for its prognosis and management. Metastatic involvement of cervical lymph nodes from oral cancer is common^(5)^. One study conducted in Pakistan showed that a majority (79%) of patients with oral cancer presented with nodal involvement and were therefore categorized as having stage 3 or 4 disease^(4)^. This underscores the importance of detecting nodal metastases in patients with oral cancer.

Metastatic involvement has a huge impact on the selection of the optimal treatment option for and determining the prognosis of patients with oral cancer^(6)^. In managing cases with cervical lymph node metastasis, the first-line option is radical or selective neck dissection, followed by chemotherapy and radiotherapy that depends on the pathological proof of nodal metastasis^(7)^. If there are clinically positive lymph nodes, the standard procedure is modified radical dissection. Aspects such as the area to be irradiated during radiotherapy may also depend on lymph node involvement. In addition, the prognosis is dependent on the presence or absence of cervical lymph node metastasis^(8)^.

Lymphadenopathy can be diagnosed by physical examination alone or by one or more forms of imaging, which can also be used for diagnostic confirmation or further characterization of the lymph nodes. Assessment of metastases in cervical lymph nodes by palpation alone has been shown to be inaccurate. When that is used as the sole method, the rate of unrecognized nodal metastases has been found to be as high as 30%^(9)^. Therefore, imaging in the form of ultrasonography or cross-sectional imaging is commonly employed for the detection and characterization of lymphadenopathy^(10)^. The criteria for pathological nodal involvement mainly involve the following aspects of the lymph node in question^(11)^: size (usually the short-axis diameter); topographic distribution; and morphological characteristics (shape, cortical thickness, and preservation or loss of fatty hila). In comparison with clinical palpation, imaging shows greater accuracy in nodal staging and can identify suspicious lymph nodes that are clinically occult. The anatomy of lymph nodes located at the retropharyngeal level make them impossible to reach by biopsy or fine-needle aspiration cytology, which makes imaging the only option for their assessment^(12)^. To avoid unnecessary surgery in patients without cervical nodal metastasis, a technique should be sensitive enough so that risk of occult metastases can be reduced to below 20%; that is, the negative predictive value should be above 80%^(13)^.

On unenhanced computed tomography scans, the density of cervical nodes is similar to that of muscle; therefore, they are delineated from the adjacent structures by their enhancement characteristics on contrast-enhanced images^(14)^. However, magnetic resonance imaging (MRI) has intrinsic good soft-tissue discrimination, making it the preferred modality for assessing the soft tissues of the oral cavity. Inclusion of advanced MRI techniques such as diffusion-weighted imaging (DWI) can improve diagnostic accuracy. In one study using histopathology as the gold standard, DWI showed 89.58% sensitivity, 76.47% specificity, and 86.15% accuracy for detecting cervical lymph node metastases^(15)^.

The objective of our study was to assess the diagnostic accuracy of DWI in detecting cervical lymph nodal metastases from oral cancer, because, to our knowledge, there have been no studies of this topic in Pakistan, as well as because the reported specificity and sensitivity of DWI in this context has varied across previous studies. If it proves to be accurate in this context, DWI will be a cost-effective means of staging oral cancer. Oral cancers have proven to be radiosensitive. Therefore, if metastases can be detected in small lymph nodes, they can be managed accordingly during the radiotherapy planning.

## MATERIALS AND METHODS

This was a cross-sectional study conducted in the Radiology Department of the Mayo Hospital, in the city of Lahore, Pakistan, from March 1st to August 31st of 2021. The study was approved by the local research ethics committee, and all participating patients gave written informed consent.

On MRI, cervical lymph nodes were considered benign if their apparent diffusion coefficient (ADC) was greater than 0.960 × 10^-3^ mm^2^/s at b-values of 600 and 1,000 s/mm^2^, whereas those with an ADC ≤ 0.960 × 10^-3^ mm^2^/s at the same b-values were considered malignant^(15)^. On histopathology, benign cervical lymph nodes were defined as those having normal cellular organization with a normal-sized nucleus and lack of pleomorphism, if there was normal mitotic activity in regional lymph nodes. Conversely, malignant cervical lymph nodes were defined as those that contained tumor cells; that is, those having abnormal cellular organization with nuclear pleomorphism and enlargement, together with increased mitotic activity in regional lymph nodes^(16)^.

The cases that tested positive for cervical lymph node metastasis by MRI and histopathology were considered true-positive cases, whereas those testing negative by both modalities were considered true-negative cases. False-positive cases were defined as those testing positive for cervical lymph node metastasis by MRI and negative by histopathology, and false-negative cases were defined as those testing negative by MRI and positive by histopathology.

The minimum sample size was calculated to be of 150 cases, with a 95% confidence level and 13% precision, on the basis of a prevalence of 30%^(15)^ with a sensitivity of 75.0%^(17)^ and a specificity of 76.47%^(17)^. Non-probability consecutive sampling was employed. The inclusion criteria were having been diagnosed with oral cancer, being between 18 and 60 years of age, and having undergone MRI at Mayo Hospital during the study period. The DWI/ADC values mentioned above were applied to those cervical lymph nodes with a short-axis diameter greater than 10 mm. Patients with contraindications to MRI (a pacemaker, clips, plates, a prosthetic valve, or any other object made of ferromagnetic material, as well as claustrophobia or an aneurysm) were excluded, as were those with a history of irradiation to the neck, those who had previously undergone surgery, and those with neck lesions not involving lymph nodes.

The study sample comprised 150 patients who presented to our radiology department with oral cancer and enlarged cervical lymph nodes on clinical examination. All of the included patients underwent MRI with a DWI as well as histopathology of cervical lymph nodes. The area included in the MRI study extended from the base of the skull to the suprasternal notch. The images were obtained in a 1.5-T scanner (Signa Voyager; GE Healthcare, Milwaukee, WI, USA) with a phased-array head and neck coil.

**Figure 1:**
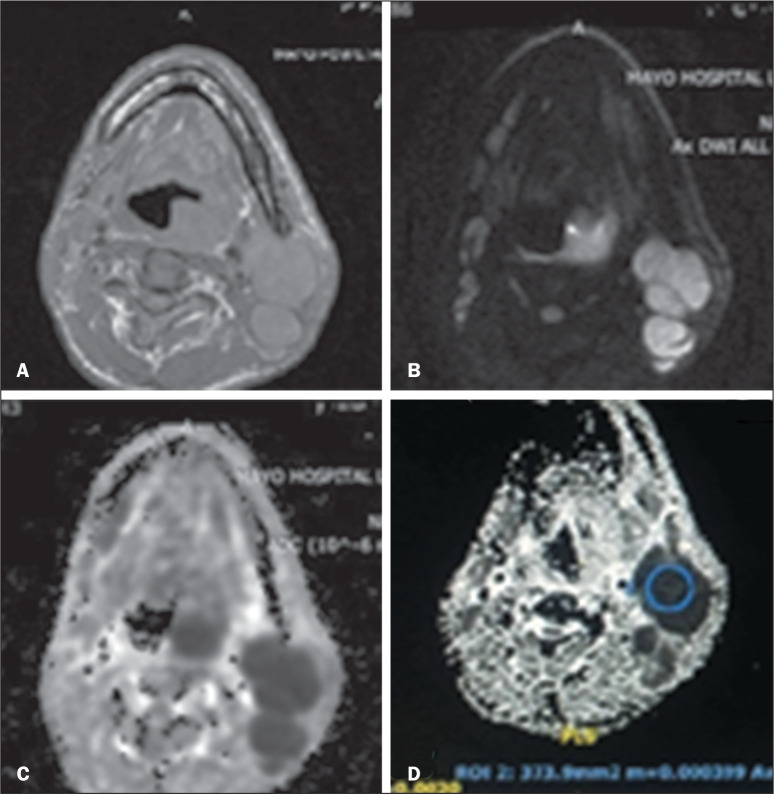
Left cervical lymph node in a patient with oropharyngeal carcinoma. The lymph node appears isointense on an axial T1-weighted image (A), bright on DWI (B) and dark on the ADC map (C). The ROI drawn on it in (D) yields an ADC value of 0.64 × 10^-3^ mm^2^/s, suggestive of metastatic involvement.

The MRI protocol was as follows: an axial T2-weighted turbo spin-echo sequence, with a repetition time/echo time (TR/TE) of 9,540/110 ms, field of view (FOV) of 240 mm, matrix of 256 × 256, and slice thickness of 4 mm; a coronal T2-weighted turbo spin-echo sequence, with a TR/TE of 7,865/124 ms, FOV of 240 mm, matrix of 320 × 320, and slice thickness of 4 mm; and an axial T1-weighted sequence, with a TR/TE of 616/85 ms, FOV of 240 mm, and slice thickness of 5 mm. For DWI, single shot spin-echo echo-planar images were obtained in the axial plane at b-values of 600 and 1,000 s/mm^2^ (TR/TE, 5,856/90 ms; matrix, 120 × 180; and slice thickness, 5 mm). The ADC maps were manipulated with the AW viewer (GE Healthcare) by drawing circular regions of interest (ROIs), which were set to be 20 mm^2^ to minimize the effect of motion artifacts in accordance with the size of the lymph node and were centered over the lymph node in question. All ADC values were obtained at b-values of 600 and 1,000 s/mm^2^ and are expressed in mm^2^/s. Figure 1 shows MRI scans with axial T1-weighted imaging, DWI, and ADC mapping with an image of ADC measurement by drawing an ROI.

Two qualified radiologists, each with at least five years of experience, evaluated the MRI scans. Both were blinded to all clinical data regarding the patients. A qualified pathologist with at least five years of experience reported the histopathology findings. Each image was assigned a diagnosis, which was then compared with the histopathological diagnosis.

The subjects were instructed to avoid swallowing during the image acquisition. As per the operational definition, nodal metastases were evaluated by MRI and histopathology. The data obtained were recorded in a pre-designed form.

### Statistical analysis

The data were analyzed with the IBM SPSS Statistics software package, version 25.0 (IBM Corp., Armonk, NY, USA). Qualitative variables are expressed as absolute values and percentages. Quantitative variables are expressed as mean and standard deviation. Diagnostic accuracy, specificity, sensitivity, positive predictive values, and negative predictive values were calculated. To deal with effect modifiers, data were stratified by age and gender.

## RESULTS

In the study population, ages ranged from 18 to 60 years, with a mean age of 41.95 ± 11.13 years. Of the 150 patients evaluated, 82 (54.67%) were between 41 to 60 years of age and 103 (46.02%) were men, with a male:female ratio of 2.2:1.

Cervical lymph node metastasis was identified on DWI in 94 (62.67%) of the patients. Histopathology findings confirmed nodal metastasis in total of 97 cases (64.67%). Among the 94 DWI-positive patients, there were 89 true-positive results and five false-positive results. Among the 56 DWI-negative patients, there were eight false-negative results and 48 true-negative results (*p* = 0.0001). The smallest cervical lymph node identified with DWI was 0.9 mm.

We found that, for the identification of cervical lymph node metastases, DWI had a sensitivity of 90.57% and a specificity of 91.75%, with positive and negative predictive values of 94.68% and 90.57%, respectively. The diagnostic accuracy of this technique for the detection of nodal metastases from oral cancer in the cervical chain was found to be 91.33% (*p* = 0.0001).

## DISCUSSION

In patients with oral cancer, nodal metastasis is associated with a poor prognosis. Although clinical palpation is performed to assess cervical lymphadenopathy, imaging is necessary to ascertain whether the lymph node enlargement is due to metastasis or to other causes, as well as to detect small lymph nodes that may not be clinically palpable^(18)^. In addition, detection of lymphadenopathy by palpation depends on the experience of the examiner, making physical palpation unreliable as a sole method for the detection of lymphadenopathy. Ultrasonography can detect the presence of lymph node enlargement but is user-dependent. It lacks the accuracy needed in order to further characterize lymphadenopathy. Cross-sectional imaging not only evaluates the extent of the primary tumor but also makes it possible to detect nodal metastases.

One study showed that differences in T1 and T2 relaxation times, which determine the signal characteristics on T1- and T2-weighted images, respectively, are not adequately reliable in differentiating between benign and malignant lymph nodes, and conventional MRI has therefore not proven to be of much value in determining nodal involvement by a tumor^(19)^. Conventional MRI relying on morphologic criteria for labeling a node as having malignant involvement has proven to have accuracy comparable to or even slightly lower than that of computed tomography, which employs similar criteria for detecting metastatic nodal involvement^(20)^. Various options are being explored to increase this accuracy, including the use of contrast agents such as ultrasmall superparamagnetic iron oxide particle contrast, which exploits microstructural, vascular, and metabolic changes induced by the tumor^(21)^. Another option is DWI, the basic principle of which is to determine differences in the random motion of water molecules in tissues, guided by variations in microstructures. Those differences are quantified by determining the ADC. The ADC represents a loss of signal when the b-value is high and has an inverse relationship with tissue cellularity^(22)^. Although some previous studies have used ADC mapping to detect nodal metastases, those studies have been focused on larger lymph nodes^(23)^. The optimum cutoff for determining whether a lymph node is benign or metastatic is also a matter of debate, with different studies using different values for making that determination.

We designed this pilot study to determine how accurate DWI is for detecting cervical lymph node metastases from oral carcinoma, using histopathology as the reference standard. As previously stated, we found that, for that purpose, DWI had an overall accuracy of 91.33%, with a sensitivity of 91.75% and a specificity of 90.57% specific for identifying metastases from oral carcinoma in cervical lymph nodes. The diagnostic accuracy was found to be 91.33%. In an earlier study, published in 2014, the overall accuracy of DWI for identifying metastases from oral carcinoma in cervical lymph nodes was found to be only 86.15%, with a sensitivity of 89.58% and a specificity of 76.47%^(15)^. In yet another study, also published in 2014, those values were 80.0%, 75.0%, and 90.9%, respectively^(17)^.

In previous studies, the reported sensitivity of DWI with ADC mapping for detecting nodal metastases has ranged from 52% to 98%, with the reported specificity ranging from 88% to 97%^(23)^. A meta-analysis of nine studies, collectively involving 337 patients, compared the ADC values of benign lymph nodes with those of metastatic lymph nodes^(24)^. The authors found that the values were markedly lower in the latter. The median ADC cutoff value applied in that study was 0.965 × 10^-3^ mm^2^/s. De Bondt et al.^(25)^ also stated that ADC criteria is the strongest independent predictor of metastases in small lymph nodes. Using DWI with ADC mapping in conjunction with other MRI sequences significantly improves the discriminating capability, with a sensitivity and specificity of 92.3% and 83.9%, respectively. Perrone et al.^(26)^ also reported a significant difference between benign and metastatic lymph nodes in terms of the ADC values. Those authors found that the mean ADC value was 0.85 × 10^-3^ mm^2^/s for the metastatic lymph nodes and 1.448 × 10^-3^ mm^2^/s for the benign lymph nodes. They determined that a cutoff value of 1.03 × 10^-3^ mm^2^/s to distinguish between benignity and malignancy was 100% sensitive and 92.9% specific. Holzapfel et al.^(27)^ applied a slightly lower ADC cutoff value (1.02 × 10^-3^ mm^2^/s) for the same purpose and obtained 94.3% accuracy, 100% sensitivity, and 87% specificity. To differentiate between metastatic lymph nodes and those with lymphomatous involvement, Zhang et al.^(28)^ recommended a cutoff ADC value of 0.77 × 10^-3^ mm^2^/s, which they found to have 83% sensitivity and 89% specificity, with an area under curve of 0.94. Wendl et al.^(29)^ conducted a similar study of patients with oral cancer and found that the best ADC cutoff value was 0.994 × 10^-3^ mm^2^/s. They found that, to identify a metastatic lymph node, that cutoff value has a sensitivity of 80%, a specificity of 65%, a positive predictive value of 31%, and a negative predictive value of 93%.

In a meta-analysis, Surov et al.^(30)^ found small sample size to be a major limiting factor in studies evaluating the utility of DWI with ADC mapping for detecting metastatic nodal involvement, resulting in a wide range of ADC values. This factor could be one of the reasons for such a large variation in the reported accuracies, sensitivities, and specificities of this technique. Another reason could be the wide range of ADC cutoff values used in various studies. Lymph node size could also be one of the factors affecting the results. The results obtained with DWI might be less reliable in terms of the visual interpretation of restricted diffusion, and the quantitative measurement could be affected by the drawing of an ROI which could be difficult to do in smaller lymph nodes. A small sample size, which can lead to underestimation or overestimation of the findings, was one of the limitations of the present study. Although previous studies have shown that pathological lymph nodes have a short-axis diameter greater than 10 mm^(11)^, the fact that we were unable to evaluate very small lymph nodes is another potential limitation of our study.

## CONCLUSION

We can conclude that DWI is highly accurate in the detection of metastatic involvement of cervical lymph nodes in patients with oral cancer. It offers a noninvasive means of detecting nodal metastases in patients with oral cancer, which is important in the staging of disease and can guide further management as well as affecting prognosis. By incorporating this technique into routine MRI performed for assessment of the extent of disease, nodal status can be determined at the same time. This can preclude the need for nodal biopsy, reducing the time to diagnosis and treatment initiation, as well as reducing patient morbidity related to complications of a biopsy.
